# Evaluation of newly proposed remission cut-points for disease activity score in 28 joints (DAS28) in rheumatoid arthritis patients upon IL-6 pathway inhibition

**DOI:** 10.1186/s13075-017-1346-5

**Published:** 2017-07-04

**Authors:** M. Schoels, F. Alasti, J. S. Smolen, D. Aletaha

**Affiliations:** 1 Second Department of Internal Medicine, Hietzing Hospital, Vienna, Austria; 2 Division of Rheumatology, Medical University of Vienna, Waehringer Guertel 18-20, 1090 Vienna, Austria

**Keywords:** Rheumatoid arthritis, DAS28, Cut-points, Outcomes research, Tocilizumab

## Abstract

**Background:**

Stringent remission criteria are crucial in rheumatoid arthritis (RA) assessment. Disease activity score in 28 joints (DAS28)-remission has not been included among American College of Rheumatology/European League Against Rheumatism definitions, because of its association with significant residual disease activity, partly due to high weighting of acute-phase reactants (APR). New, more stringent cut-points for DAS28-remission have recently been proposed that are suggested to reflect remission by clinical and simplified disease activity indices (clinical disease activity index (CDAI), simple disease activity index (SDAI)). However, their stringency in therapies directly influencing APR, like IL-6-blockers, has not been tested. We tested the new cut-points in patients with RA receiving tocilizumab.

**Methods:**

We used data from randomised controlled trials of tocilizumab and evaluated patients in remission according to new DAS28-C-reactive protein (DAS-CRP) and DAS-erythrocyte sedimentation rate (DAS-ESR) cut-points (1.9 and 2.2). We assessed their disease activity state using the CDAI, SDAI and Boolean criteria and analysed their individual residual core set variables, like swollen joint counts (SJC28).

**Results:**

About 50% of patients in DAS28-CRP-remission (<1.9) fell into higher disease activity states when assessed with CDAI, SDAI or Boolean criteria. Also, 15% had three or more (up to eight) SJC. Even higher disease activity was seen in patients classified as being in DAS28-ESR-remission (<2.2).

**Conclusions:**

Even with new, more stringent cut-points, DAS28-remission is frequently associated with considerable residual clinical disease activity, indicating that this limitation of the DAS28 is related to score construction rather than the choice of cut-points.

**Electronic supplementary material:**

The online version of this article (doi:10.1186/s13075-017-1346-5) contains supplementary material, which is available to authorized users.

## Background

Composite measures to define disease activity provide better information than individual variables in the assessment of rheumatoid arthritis (RA) [1]. Among these instruments are dichotomous tools like the American College of Rheumatology (ACR) response criteria, [2] and continuous scores like the simplified and clinical disease activity indices (SDAI and CDAI) [3, 4] and the disease activity score using 28 joint counts (DAS28) in its two versions employing erythrocyte sedimentation rate (ESR) or C-reactive protein (CRP) [5]. The use of continuous measures to assess disease activity states is an important requirement in clinical trials and practice, and achieving a state of low disease activity (LDA) or remission (REM) is a major treatment target in RA [6]. Consequently, stringent remission definitions are crucial for optimising outcomes [7].

DAS28-remission has not been included among the joint remission definitions by the ACR and the European League Against Rheumatism (EULAR), because it is associated with significant residual disease activity in a large proportion of patients [7–9]. Due to the high weight of acute phase reactant (APR) components in the DAS28 formula, this impediment becomes particularly prominent when agents that interfere directly with the acute-phase response, like the interleukin-6 (IL-6) pathway inhibitors or Janus kinase (Jak) inhibitors, are used. Recognizing this limitation of the DAS28, which is not seen with the CDAI and SDAI [10], cut-points other than 2.6 have been proposed [11–13]. The most recent approach suggested a DAS28-CRP <1.9 and DAS28-ESR <2.2 to be related best to CDAI-remission [13]. However, survey results show that remission should define a state of at most minimal residual disease activity with no more than two involved joints, swollen and/or tender [7]. As reported previously, upon IL-6 pathway inhibition low APR levels lead to unduly high remission frequencies as assessed by the DAS28 [10] while at the same time allowing for a significant number of residual swollen joints.

Here, we tested the newly proposed cut-points for DAS28-CRP and DAS28-ESR remission in RA patients treated with tocilizumab (TCZ), an approved and widely used antibody to the IL-6 receptor.

## Methods

### Data

We analysed data from three large, randomized, controlled trials. The LITHE, [14] OPTION, [15] and TOWARD [16] studies evaluated the efficacy of TCZ plus methotrexate/conventional synthetic disease-modifying anti-rheumatic drugs (csDMARDs) in adult patients with RA with prior non-response to csDMARDs. We were kindly provided with a random 80% data cut by the trial sponsor (Roche). For our analyses, we used data from the TCZ treatment arms, i.e., patients receiving 4 and 8 mg/kg intravenous TCZ in combination with methotrexate or csDMARDs.

### Analyses

We examined disease activity parameters in patients who were classified as being in either DAS28-CRP (<1.9) or DAS28-ESR (<2.2) remission after 24 weeks follow up. We analysed levels of core set variables used for calculation of composite scores (formulas compiled in Additional file 1: Table S1), namely SJC/tender joint count (TJC) using 28 joints, patient and evaluator global assessment (PGA, EGA) and CRP and ESR. We contrasted remission according to newly proposed DAS28 cut-points with percentages of REM, LDA, moderate disease activity (MDA) and high disease activity (HDA) using the CDAI [4] or SDAI [3] and also tested Boolean remission criteria [7]. Cut-points for the CDAI (SDAI) were applied as follows: REM ≤2.8 (≤3.3), LDA >2.8 and ≤10 (>3.3 and ≤11), and MDA >10 and ≤22 (>11 and ≤26).

## Results

In total, 2423 patients (TCZ: 1613, placebo: 810) were included in the LITHE, OPTION and TOWARD studies (Additional file 1: Table S2). After 24 weeks of TCZ treatment, 178 patients achieved remission according to the DAS28-CRP <1.9 threshold. Additional file 1: Table S3 shows their baseline characteristics; briefly, mean CRP was 2.6 mg/dl, mean ESR was 46 mm/h, and mean SJC28 was 10.8. Baseline composite scores in DAS28-CRP-remission were: DAS28-CRP 5.6, DAS28-ESR 6.2, SDAI 37.6 and CDAI 35.0. Baseline characteristics of the 235 patients with DAS28-ESR <2.2 at week 24 are also detailed in Additional file 1: Table S3.

### Higher remission rates by DAS28 compared to CDAI or SDAI

Among patients in DAS28-CRP-remission, only 47.2% were in remission according to the CDAI while 52.8% were in LDA. SDAI evaluation resulted in 52.8% in REM, 47.2% in LDA and 39.3% in REM according to Boolean criteria (Additional file 1: Table S4). Among the DAS28-ESR remitters, only 30.2% reached CDAI-REM, while 60.9% were in CDAI-LDA, and 8.9% were even in CDAI-MDA (SDAI: 34.5% in REM, 58.3% in LDA, and in 7.2% MDA; Boolean criteria: 24.3% in REM; Additional file 1: Table S4).

### Individual disease activity parameters in DAS28-remission

**Fig. 1:**
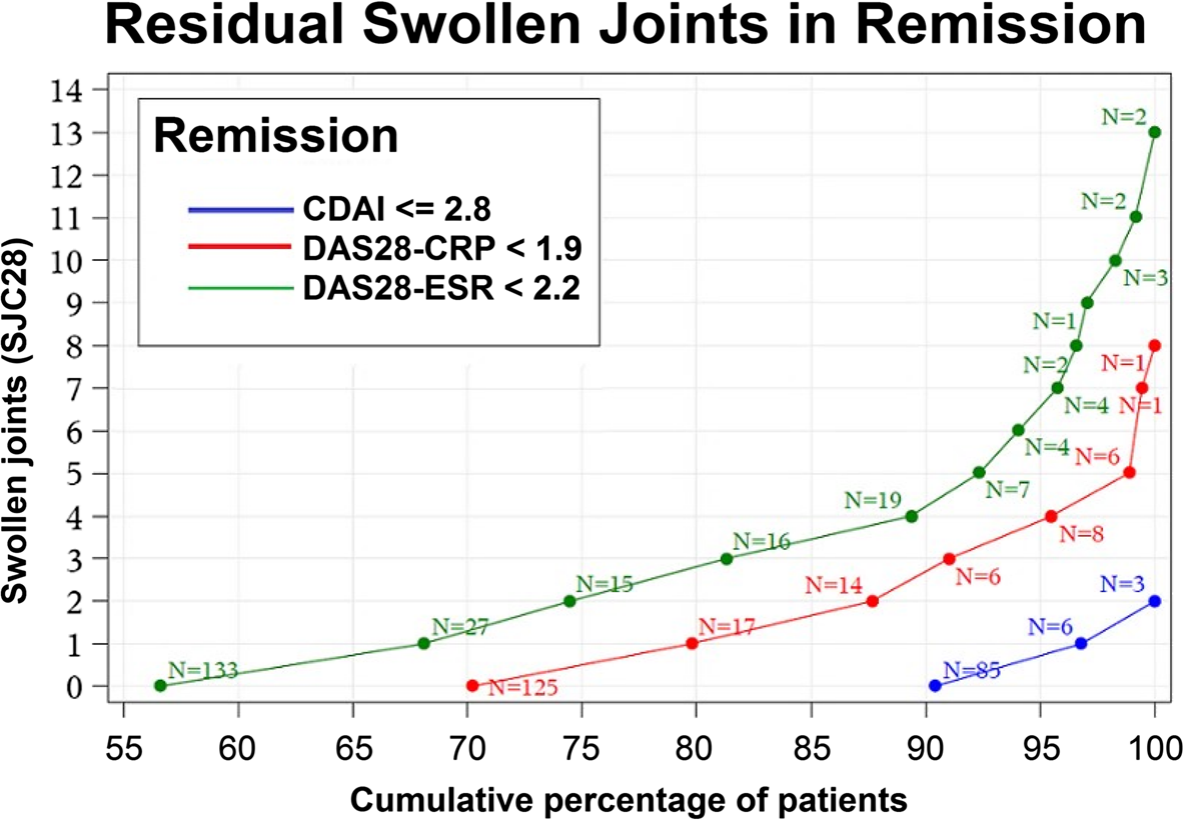
Residual swollen joints in remission. *X-axis* shows cumulative percent of patients. *Y-axis* shows swollen joint counts (SJC28). *Red line*: counts of patients in remission according to DAS28-CRP (DAS28-CRP <1.9; N = 178); *green line*: remission according to DAS28-ESR (DAS28-ESR <2.2; N = 235); *blue line*: remission according to CDAI (CDAI ≤2.8; N = 94)

We analysed individual disease activity parameters in patients who reached DAS28-REM at 24 weeks (Table 1). We found that using the SJC in 28 joints (SJC28), up to 8 patients were in DAS28-CRP-REM, and up to 13 were in DAS28-ESR-REM. Figure 1 shows residual numbers of SJC in patients in DAS28-CRP and DAS28-ESR vs. patients in CDAI remission. Almost 15% of remitters had at least three swollen joints; among the 10% of patients with the highest SJC28, the mean SJC was 4.3 (Additional file 1: Table S5), which was made possible by very low APR, i.e., mean CRP of 0.03 mg/dl and mean ESR of 4.2 mm/h. Only 39.3% of patients with DAS28-CRP <1.9 and 24.3% with DAS28-ESR <2.2 fulfilled the Boolean remission definition.

**Table 1:** Disease activity parameters of patients in DAS28-CRP or DAS28-ESR remission, but not in SDAI, CDAI or Boolean remission

	DAS28-CRP <1.9 (n = 178)				DAS28-ESR <2.2 (n = 235)			
	No CDAI REM (n = 94)	No SDAI REM (n = 84)	No Boolean REM (n = 108)	All (n = 178)	No CDAI REM (n = 164)	No SDAI REM (n = 154)	No Boolean REM (n = 178)	All (n = 235)
SJC28	1.3 (1.9)	1.5 (1.9)	1.2 (1.8)	0.8 (1.5)	2.2 (2.8)	2.3 (2.9)	2.0(2.8)	1.6 (2.6)
	0 (0.0–8.0)	0 (0.0–8.0)	0.0 (0.0–8.0)	0.0 (0.0–8.0)	1.0 (0.0–13.0)	1.0 (0.0–13.0)	1.0 (0.0–13.0)	0.0 (0.0–13.0)
TJC28	0.1 (0.3)	0.1 (0.2)	0.0 (0.2)	0.1 (0.3)	0.8 (1.3)	0.8 (1.2)	0.7 (1.1)	0.6 (1.0)
	0 (0.0–1.0)	0 (0.0–1.0)	0.0 (0.0–1.0)	0.0 (0.0–1.0)	0.0 (0.0–5.0)	0.0 (0.0–5.0)	0.0 (0.0–5.0)	0.0 (0.0–5.0)
CRP (mg/l)	0.6 (0.8)	0.7 (0.9)	0.7 (0.9)	0.7 (1.0)	1.4 (4.1)	1.4 (4.2)	1.7 (4.8)	1.5 (4.2)
	0.3 (0.2–5.1)	0.3 (0.2–5.1)	0.4 (0.2–5.2)	0.3 (0.2–7.1)	0.4 (0.2–39.7)	0.4 (0.2–39.7)	0.4 (0.2–39.7)	0.4 (0.2–39.7)
ESR (mm/h)	8.4 (10.6)	8.5 (9.8)	8.4 (10.3)	9.3 (12.4)	3.6 (3.1)	3.7 (3.1)	3.9 (3.3)	4.2 (3.5)
	4.0 (0.0–57.0)	5.0 (0.0–45.0)	5.0 (0.0–57.0)	5.0 (.00–91)	3.0 (1.0–16.0)	3.0 (1.0–16.0)	3.0 (1.0–18.0)	3.0 (1.0–18.0)
PGA (mm VAS)	20.4 (14.8)	21.7 (15.2)	20.2 (13.9)	13.7 (13.7)	21.9 (17.8)	22.9 (18.1)	21.4 (17.4)	17.1 (17.0)
	16.0 (0.0–61.0)	19.0 (0–61.0)	16.0 (0.0–61.0)	10.0 (0.0–61.0)	17.5 (0.0–89.0)	19.5 (0.0–89.0)	16.5 (0.0–89.0)	13.0 (0.0–89.0)
EGA (mm VAS)	14.6 (11.4)	15.4 (12.0)	12.2 (11.5)	9.8 (10.4)	14.3 (11.4)	14.7 (11.7)	13.0 (11.5)	11.4 (10.9)
	12.0 (0.0–63.0)	13.0 (0–63.0)	9.0 (0.0–63.0)	7.0 (0.0–63.0)	11.5 (0.0–75.0)	12.0 (0.0–75.0)	10.0 (0.0–75.0)	9.0 (0.0–75.0)
HAQ	0.7 (0.6)	0.7 (0.6)	0.7 (0.6)	0.6 (0.5)	0.7 (0.6)	0.7 (0.6)	0.7 (0.6)	0.6 (0.6)
	0.6 (0.0–2.0)	0.6 (0–2.0)	0.6 (0.0–2.0)	0.4 (0.0–2.0)	0.6 (0.0–2.3)	0.6 (0.0–2.3)	0.6 (0.0–2.3)	0.5 (0.0–2.3)
SDAI	5.0 (1.6)	5.3 (1.5)	4.6 (1.9)	3.3 (2.2)	6.7 (3.3)	7.0 (3.2)	6.3 (3.5)	5.2 (3.7)
	4.7 (2.8–9.5)	4.9 (3.3–9.5)	4.4 (1.3–9.5)	3.1 (0.02–9.5)	5.8 (2.8–19.6)	6.1 (3.4–19.6)	5.6 (1.3–19.6)	4.6 (0.02–19.6)
CDAI	4.9 (1.6)	5.2 (1.5)	4.5 (1.9)	3.2 (2.3)	6.6 (3.3)	6.8 (3.2)	6.2 (3.5)	5.0 (3.7)
	4.6 (2.8–9.5)	4.8 (2.9–9.5)	4.4 (1.2–9.5)	3.0 (0.0–9.5)	5.8 (2.8–19.6)	6.1 (3.2–19.6)	5.5 (0.0–19.6)	4.5 (0.0–19.6)
DAS28–CRP	1.7 (0.2)	1.7 (0.2)	1.6 (0.2)	1.5 (0.3)	2.1 (0.5)	2.1 (0.5)	2.0 (0.5)	1.9 (0.6)
	1.7 (1.1–1.9)	1.7 (1.1–1.9)	1.7 (1.2–1.9)	1.6 (1.0–1.9)	2.0 (1.1–3.7)	2.0 (1.1–3.7)	2.0 (1.2–3.7)	1.8 (1.0–3.7)
DAS28–ESR	1.7 (0.7)	1.7 (0.7)	1.6 (0.7)	1.5 (0.8)	1.6 (0.5)	1.6 (0.5)	1.6 (0.5)	1.5 (0.5)
	1.6 (0.4–3.4)	1.7 (0.5–3.4)	1.6 (0.2–3.4)	1.5 (0.0–3.4)	1.7 (0.4–2.2)	1.8 (0.5–2.2)	1.7 (0.2–2.2)	1.6 (0.0–2.2)

Values are mean (SD) or median (range). *SDAI* simplified disease activity index *CDAI* clinical disease activity index, *REM* remission, *SJC28* swollen joint count using 28 joints, *TJC28* tender joint count using 28 joints, *CRP* C-reactive protein, *ESR* erythrocyte sedimentation rate, *PGA* patient global assessment, *VAS* visual analogue scale, *EGA* evaluator global assessment, *HAQ* health assessment questionnaire, *DAS28-CRP* disease activity score using 28 joint counts and C-reactive protein, *DAS28-ESR* disease activity score using 28 joint counts and erythrocyte sedimentation rate

We obtained very similar results for DAS28-ESR-REM regarding number of residual swollen joints (data not shown). Among patients who met DAS28-ESR-remission criteria, but not those for the CDAI, the mean SJC28 was 2.2 (2.8) (Table 1). More than 10% had five or more and >25% had three or more swollen joints. APR in the 10% with the highest number of swollen joints averaged at 0.08 (0.12) mg/dl (CRP) and 2.3 (1.5) mm/h (ESR) (Additional file 1: Table S5).

In addition, DAS28-remission allowed for a patient global assessment of disease activity (PGA) of up to 61 mm (DAS28-CRP-REM), and 89 mm (DAS28-ESR-REM) on the 100 mm visual analogue scale; the evaluator global assessment (EGA) reached up to 63 mm in DAS28-CRP-REM and 75 mm in DAS28-ESR-REM (Table 1). Importantly, this was not the case with the CDAI and SDAI ratings. Additional file 2: Figure S1 displays individual disease activity parameters that contribute to the calculated composite scores for patients in DAS28-CRP-remission (panel A), and in DAS28-ESR-remission (panel B). As can be seen, in DAS28-remitters, who were in LDA or MDA according to the CDAI, lower APR “compensated” for higher joint counts, allowing maintenance of DAS28-remission despite lower cut-points.

## Discussion

In 2011, the ACR and EULAR provided Boolean and index-based remission criteria for trials and clinical practice, implementing survey results and analyses of radiographic and functional outcomes [7]. At that time, the DAS28 remission criteria were not compatible with these important constructs and outcomes. In the meantime, new lower cut-points of 1.9 for DAS28-CRP-remission and 2.2 for DAS28-ESR-remission have been proposed [13].

In the present study, we observed that these cut-points still allow a considerable proportion of patients with RA to be classified as remitters despite the presence of a significant SJC, namely up to 8 in DAS28-CRP remission and 13 in DAS28-ESR remission. These numbers do not represent individual outliers, as approximately 15% and 25%, respectively, of patients in putative remission according to the proposed thresholds had three or more swollen joints.

The SJC is highly related to the progression of joint damage [17], therefore any remission criteria allowing for swollen joints in a substantial number of patients would not pass this important filter of criterion validity, and would not have face validity for most rheumatologists [18]. A majority of patients with DAS28-CRP <1.9 were in LDA according to the CDAI, and about two thirds of patients with DAS28-ESR <2.2 were not in remission as defined by the CDAI, with almost 10% even being in CDAI-MDA. High SJCs were not an isolated finding but rather accompanied by higher PGA score, pain and EGA ratings and worse function. However, CRP or ESR was lower among DAS28 “remitters” who had CDAI LDA or MDA. Thus, in the formula of DAS28, very low APR within the normal range may compensates for unacceptably high joint counts.

Our results suggest that the problem of DAS28-remission is not related to a specific cut-point, but rather to the construction of the score itself: the complexity, transformations and weighting of the formula will perpetuate the problem, even if the cut-point is dramatically reduced. Indeed, one could have envisaged that lower cut-points would not be the solution as the ACR/EULAR task force had tested a DAS28-ESR threshold of 2.0 and did not find it compatible with optimal outcomes [7]. Also, there was no major difference in sonographic data between cut-points of 2.6 and 2.4 [19]. At the time the DAS28 was introduced, it was a seminal approach to assess disease activity, but remission was only rarely achievable and the weighting of the individual score components was appropriate for higher disease activity states.

When we carried out these analyses for the SDAI, which also includes CRP in its formula, we found remission rates resembling those of the CDAI, a purely clinical score, more closely than those of the DAS28. However, the contribution of CRP to the SDAI only amounts to about 5% [4]. These results emphasise further that not the mere presence but the high weighting of APR in the DAS28 formula may lead to misrepresentation of actual disease activity.

Interestingly, Nishimoto and colleagues conducted correlation analyses between CDAI and DAS28-ESR in 53 patients included in the SATORI study at baseline and follow up [20]. They observed strong correlation between the DAS8 and the CDAI or SDAI and concluded that the DAS28-ESR was a valid tool to assess patients treated with TCZ; nevertheless, this correlation only addressed the relationships between the scores for higher and lower disease activity, which will be found for most scores, and do not provide a comparative answer in the clinical context. In addition, they also reported a threefold difference between rates of DAS28-remission and CDAI-remission after 24 weeks (with the traditional thresholds for DAS28-ESR of <2.6). Finally, patients who were DAS28-ESR remitters but not CDAI/SDAI remitters (*n* = 17) had high residual swollen joints and/or PGA; indeed, among DAS28-remitters, only 44% had no swollen joints, while among SDAI and CDAI remitters almost 90% had no swollen joints. Thus, their data fully support our general assessment.

Also Shaver et al. [21] investigated remission rates using different methods of assessment. In their cross-sectional analysis of data from an outpatient clinic, the authors included RA patients on various therapies, which were partly csDMARDs and partly biological agents, with no further specification. In this cohort, the authors identified a similar if not even greater discrepancy among remission rates: specifically, the prevalence of remission differed dramatically between the scores (28.5% when using the DAS28 compared to 6.5–8.1% when using the CDAI). Thus, these data also support concerns about the high weighting of APRs in the DAS28 formulas, which we have now also shown to affect the DAS28 regardless of the new (lower) thresholds.

Our study has some limitations. First, it focussed on TCZ data only. However, we have previously shown that DAS28-CRP is also not a reliable instrument for the assessment of remission in tofacitinib therapy [22]. Data on sarilumab, sirukumab and baricitinib need to be obtained to validate the current findings. Second, we did not evaluate radiographic changes. However, when biologic agents are used, we cannot expect joint damage progression even in active disease [23], and the number of placebo-treated patients in remission was very small. Moreover, it has previously been shown that in DAS28 but not in SDAI or CDAI remission, it is mainly the SJC that drives the assessment of joint deterioration, [24] in line with findings that in individual joints swelling is highly related to damage [17].

## Conclusions

Lower more stringent remission thresholds for DAS28, as a consequence of the construction of the formula, do not convey sufficient stringency to comply with full clinical remission defined as a state of no or at most minimal disease activity. If one patient in seven to one patient in four who are categorized as being in “remission” has three or more swollen joints, full clinical remission cannot be claimed. Our data further confirm the validity of the ACR/EULAR remission definition, especially when agents interfering directly with APR are employed.

## Additional files

Additional file 1: **Table S1.** Formulas of compound scores for RA assessment. **Table S2.** Baseline characteristics of the LITHE, OPTION and TOWARD trial data provided for the present analyses. **Table S3.** Baseline characteristics of patients achieving DAS28-CRP or DAS28-ESR remission at week 24: investigated cohort. **Table S4.** Comparison of remission rates according to different compound scores (24-week values). **Table S5.** Mean values of core set variables and composite measures in the 10% of patients with the highest swollen joint counts in DAS28-CRP and DAS28-ESR remission (90^th^ percentile of SJC). (DOCX 22 kb)

Additional file 2: **Figure S1.** Display of individual disease activity parameters among patients with DAS28-CRP<1.9 who attain different states by CDAI after 24 weeks of tocilizumab therapy. Panel a: patients in remission according to DAS28-CRP classification (n=178). Panel b: patients in remission according to DAS28-ESR classification (n=235). X-axis: CDAI disease activity state. Y-axis: Mean values of respective disease activity parameters. (TIF 2475 kb)
