# Supplementary material for: Evaluation of newly proposed remission cut-points for disease activity score in 28 joints (DAS28) in rheumatoid arthritis patients upon IL-6 pathway inhibition
Source: Arthritis Res Ther. 2017 Jul 4;19:155. doi: 10.1186/s13075-017-1346-5 (PMC5496440; doi:10.1186/s13075-017-1346-5)
Supplement: Supplementary file 1 — Table S1. Formulas of compound scores for RA assessment. Table S2. Baseline characteristics of the LITHE, OPTION and TOWARD trial data provided for the present analyses. Table S3. Baseline characteristics of patients achieving DAS28-CRP or DAS28-ESR remission at week 24: investigated cohort. Table S4. Comparison of remission rates according to different compound scores (24-week values). Table S5. Mean values of core set variables and composite measures in the 10% of patients with the highest swollen joint counts in DAS28-CRP and DAS28-ESR remission (90th percentile of SJC). (DOCX 22 kb) [file 13075_2017_1346_MOESM1_ESM.docx]

# Additional file 1

# **Evaluation of Newly Proposed Remission Cut-points for DAS28 in Rheumatoid Arthritis Patients upon IL-6 Pathway Inhibition**

*including Tables S1, S2, S3, S4 and S5.*

## Table S1. Formulas of compound scores for RA assessment

| **Score** | **Formula** |
| --- | --- |
| **CDAI** | SJC28 + TJC28 + PGA + EGA |
| **SDAI** | SJC28 + TJC28 + PGA + EGA + CRP |
| **DAS28-CRP** | 0.56*√TJC28 + 0.28*√SJC28 + 0.36*ln(CRP+1) + 0.014*PGA + 0.96 |
| **DAS28-ESR** | 0.56*√TJC28 + 0.28*√SJC28 + 0.70*ln(ESR) + 0.014*PGA |
| **Boolean Criteria** | SJC≤1, TJC≤1, PGA≤1cm, CRP≤1mg/dl |

**Table S1.** CDAI=clinical disease activity index; SDAI=simplified disease activity index; DAS28-CRP=disease activity score using 28 joint counts and C-reactive protein; DAS28-ESR= disease activity score using 28 joint counts and erythrocyte sedimentation rate; SJC28=swollen joint count using 28 joints; TJC28=tender joint count using 28 joints; CRP=C-reactive protein; ESR=erythrocyte sedimentation rate; PGA=patient global assessment (visual analogue scale; VAS 0-10cm for CDAI and SDAI; 0-100mm VAS for DAS28); EGA=evaluator global assessment (0-10cm VAS).

## Table S2. Baseline characteristics of the LITHE, OPTION and TOWARD trial data provided for the present analyses

| **Variable Name** | **LITHE** | | **OPTION** | | **TOWARD** | **Pooled treatment groups** | **Pooled placebo groups** | **ALL** |
| --- | --- | --- | --- | --- | --- | --- | --- | --- |
|  | 8mg TCZ | 4 mg TCZ | 8mg TCZ | 4mg TCZ | 8mg TCZ |  |  |  |
| **Patient N** | 321 | 317 | 160 | 170 | 645 | 1613 | 810 | 2423 |
| **Age [years]** | 53.8(11.6) | 51.8(12.6) | 50.7(11.8) | 51.5(12.7) | 52.9(12.6) | 52.5(12.4) | 51.9(12.7) | 52.3(12.5) |
| **Female [%]** | 83.2 | 84.5 | 84.4 | 80.0 | 81.4 | 82.5 | 82.4 | 82.5 |
| **Disease duration [years]** | 9.7(8.5) | 9.4(7.6) | 7.4(7.2) | 7.6(7.2) | 9.8(8.8) | 9.2(8.3) | 9.0(8.3) | 9.1(8.3) |
| **HAQ** | 1.5(0.6) | 1.5(0.7) | 1.6(0.6) | 1.7(0.6) | 1.5(0.6) | 1.5(0.6) | 1.6(0.6) | 1.5(0.6) |
| **PGA [mm VAS]** | 61.9(22.0) | 60.2(23.8) | 65.9(22.4) | 65.7(20.4) | 66.5(22.3) | 64.2(22.5) | 64.7(23.1) | 64.4(22.7) |
| **EGA [mm VAS]** | 62.1(17.0) | 62.1(16.9) | 64.5(15.1) | 64.1(15.6) | 64.0(16.5) | 63.3(16.5) | 63.1(16.9) | 63.2(16.6) |
| **PP [mm VAS]** | 55.0(21.6) | 52.2(21.9) | 60.7(22.6) | 60.4(21.0) | 58.5(22.3) | 57.0(22.2) | 57.1(22.5) | 57.0(22.3) |
| **CRP [mg/dl]** | 2.2(2.3) | 2.1(2.4) | 2.8(2.7) | 2.9(3.5) | 2.6(3.3) | 2.5(2.9) | 2.4(2.8) | 2.5(2.9) |
| **ESR [mm/h]** | 46.7(25.6) | 47.2(25.4) | 50.4(25.8) | 48.3(26.0) | 48.3(28.1) | 48.0(26.7) | 48.6(26.9) | 48.2(26.8) |
| **SCJ28** | 12.0(5.7) | 12.2(5.6) | 13.4(5.9) | 13.2(6.0) | 13.2(6.4) | 12.8(6.0) | 12.5(5.7) | 12.7(5.9) |
| **TJC28** | 15.5(6.7) | 15.6(6.7) | 17.6(6.7) | 17.4(6.9) | 16.2(7.3) | 16.2(7.0) | 15.8(7.0) | 16.1(7.0) |
| **SDAI** | 42.0(12.9) | 42.2(12.7) | 46.8(13.8) | 46.5(12.8) | 45.1(14.6) | 44.2(13.8) | 43.6(13.4) | 44.0(13.7) |
| **CDAI** | 39.7(12.4) | 40.1(12.1) | 44.0(13.2) | 43.6(12.5) | 42.5(14.0) | 41.7(13.2) | 41.2(12.6) | 41.5(13.0) |
| **DAS28-ESR** | 6.5(1.0) | 6.5(0.9) | 6.9(0.9) | 6.8(0.9) | 6.7(1.0) | 6.7(1.0) | 6.6(1.0) | 6.7(1.0) |
| **DAS28-CRP** | 5.9(0.9) | 5.9(0.9) | 6.2(0.9) | 6.2(0.9) | 6.1(1.0) | 6.0(1.0) | 6.0(1.0) | 6.0(1.0) |

**Table S2.** HAQ=health assessment questionnaire; PGA=patient global assessment; VAS=visual analogue scale; EGA=evaluator global assessment; PP=patient pain assessment; CRP=C-reactive protein; ESR=erythrocyte sedimentation rate; SJC28=swollen joint count using 28 joints; TJC28=tender joint count using 28 joints. Values in mean (SD) if not stated otherwise.

## Table S3. Baseline characteristics of patients achieving DAS28-CRP or DAS28-ESR remission at week 24: investigated cohort

|  | **DAS28-CRP<1.9** | **DAS28-ESR<2.2** |
| --- | --- | --- |
| **Patient N** | 178 | 235 |
| **Age [years]** | 53.3(13.7) | 52.7(13.6) |
| **Female [%]** | 77.5 | 76.2 |
| **Disease duration [years]** | 9.1(9.8) | 8.8(8.7) |
| **HAQ** | 1.3(0.6) | 1.3(0.6) |
| **PGA [mm VAS]** | 57.7(23.5) | 58.0(23.0) |
| **EGA [mm VAS]** | 57.6(17.0) | 58.4(16.7) |
| **PP [mm VAS]** | 48.1(22.7) | 50.8(22.9) |
| **CRP [mg/dl]** | 2.6(3.1) | 2.4(2.3) |
| **ESR [mm/h]** | 46.0(27.9) | 39.2(24.0) |
| **SCJ28** | 10.8(5.8) | 11.2(5.7) |
| **TJC28** | 12.8(6.6) | 13.2(6.6) |
| **SDAI** | 37.6(13.7) | 38.5(13.0) |
| **CDAI** | 35.0(12.9) | 36.1(12.5) |
| **DAS28-ESR** | 6.2(1.0) | 6.1(1.0) |
| **DAS28-CRP** | 5.6(1.0) | 5.7(1.0) |

**Table S3.** HAQ=health assessment questionnaire; PGA=patient global assessment; VAS=visual analogue scale; EGA=evaluator global assessment; PP=patient pain assessment; CRP=C-reactive protein; ESR=erythrocyte sedimentation rate; SJC28=swollen joint count using 28 joints; TJC28=tender joint count using 28 joints. Values in mean (SD) if not stated otherwise.

## Table S4. Comparison of remission rates according to different compound scores (24-week values)

|  | **CDAI** | | | | **SDAI** | | | | **Boolean Criteria** | |
| --- | --- | --- | --- | --- | --- | --- | --- | --- | --- | --- |
|  | %REM | %LDA | %MDA | %HDA | %REM | %LDA | %MDA | %HDA | %REM | %NON-REM |
| **DAS28-CRP<1.9 remission; N=178** | 47.2 | 52.8 | 0 | 0 | 52.8 | 47.2 | 0 | 0 | 39.3 | 60.7 |
| **DAS28-ESR <2.2 remission; N=235** | 30.2 | 60.9 | 8.9 | 0 | 34.5 | 58.3 | 7.2 | 0 | 24.3 | 75.7 |

**Table S4.** SDAI=simplified disease activity index; CDAI=clinical disease activity index; REM=remission; LDA=low disease activity; MDA=moderate disease activity; HDA=high disease activity; DAS28-CRP=Disease activity score using 28 joints and C-reactive protein.

## Table S5. Mean values of core set variables and composite measures in the 10% of patients with the highest swollen joint counts in DAS28-CRP and DAS28-ESR remission (90^th^ Percentile of SJC)

|  | **DAS28-CRP < 1.9**  **N=22** | | **DAS28-ESR < 2.2**  **N=25** | |
| --- | --- | --- | --- | --- |
|  | **Mean (SD)** | **Median (range)** | **Mean (SD)** | **Median (range)** |
| **SCJ28** | 4.3 (1.3) | 4.0 (3.0-8.0) | 7.6 (2.6) | 7.0 (5.0-13.0) |
| **TJC28** | 0.0 (0.0) | 0.0 (0.0-0.0) | 0.3 (0.6) | 0.0 (0.0-2.0) |
| **CRP [mg/dl]** | 0.03 (0.02) | 0.02 (0.02-0.07) | 0.08 (0.12) | 0.04 (0.02-0.61) |
| **ESR [mm/h]** | 4.2 (4.5) | 3.0 (0.0-18.0) | 2.3 (1.5) | 2.0 (1.0-6.0) |
| **PGA [mm VAS]** | 6.8 (5.2) | 6.0 (0.0-15.0) | 14.4 (20.6) | 7.0 (0.0-89.0) |
| **EGA [mm VAS]** | 11.6 (11.7) | 5.5 (1.0-50.0) | 15.3 (16.4) | 10.0 (1.0-75.0) |
| **Pain [mm VAS]** | 6.1 (6.3) | 3.0 (0-20.0) | 13.5 (20.6) | 5.0 (0.0-78.0) |
| **HAQ** | 0.4 (0.5) | 0.2 (0.0-1.4) | 0.5 (0.6) | 0.3 (0.0-1.9) |
| **SDAI** | 6.2 (1.3) | 5.9 (4.5-9.3) | 11.0 (4.4) | 9.1 (5.5-19.6) |
| **CDAI** | 6.2 (1.3) | 5.9 (4.4-9.3) | 10.9 (4.4) | 9.0 (5.5-19.6) |
| **DAS28-CRP** | 1.7 (0.1) | 1.7 (1.5-1.9) | 2.3 (0.5) | 2.1 (1.7-3.7) |
| **DAS28-ESR** | 1.5 (0.6) | 1.4 (0.6-2.7) | 1.6 (0.5) | 1.5 (0.7-2.2) |

**Table S5.** DAS28-CRP=disease activity score using 28 joint counts and C-reactive protein; DAS28-ESR= disease activity score using 28 joint counts and erythrocyte sedimentation rate; SJC28=swollen joint count using 28 joints; TJC28=tender joint count using 28 joints; CRP=C-reactive protein; ESR=erythrocyte sedimentation rate; PGA=patient global assessment; VAS=visual analogue scale; EGA=evaluator global assessment; HAQ=health assessment questionnaire.
