# Supplementary figures and images for: Evaluation of newly proposed remission cut-points for disease activity score in 28 joints (DAS28) in rheumatoid arthritis patients upon IL-6 pathway inhibition
Source: Arthritis Res Ther. 2017 Jul 4;19:155. doi: 10.1186/s13075-017-1346-5 (PMC5496440; doi:10.1186/s13075-017-1346-5)

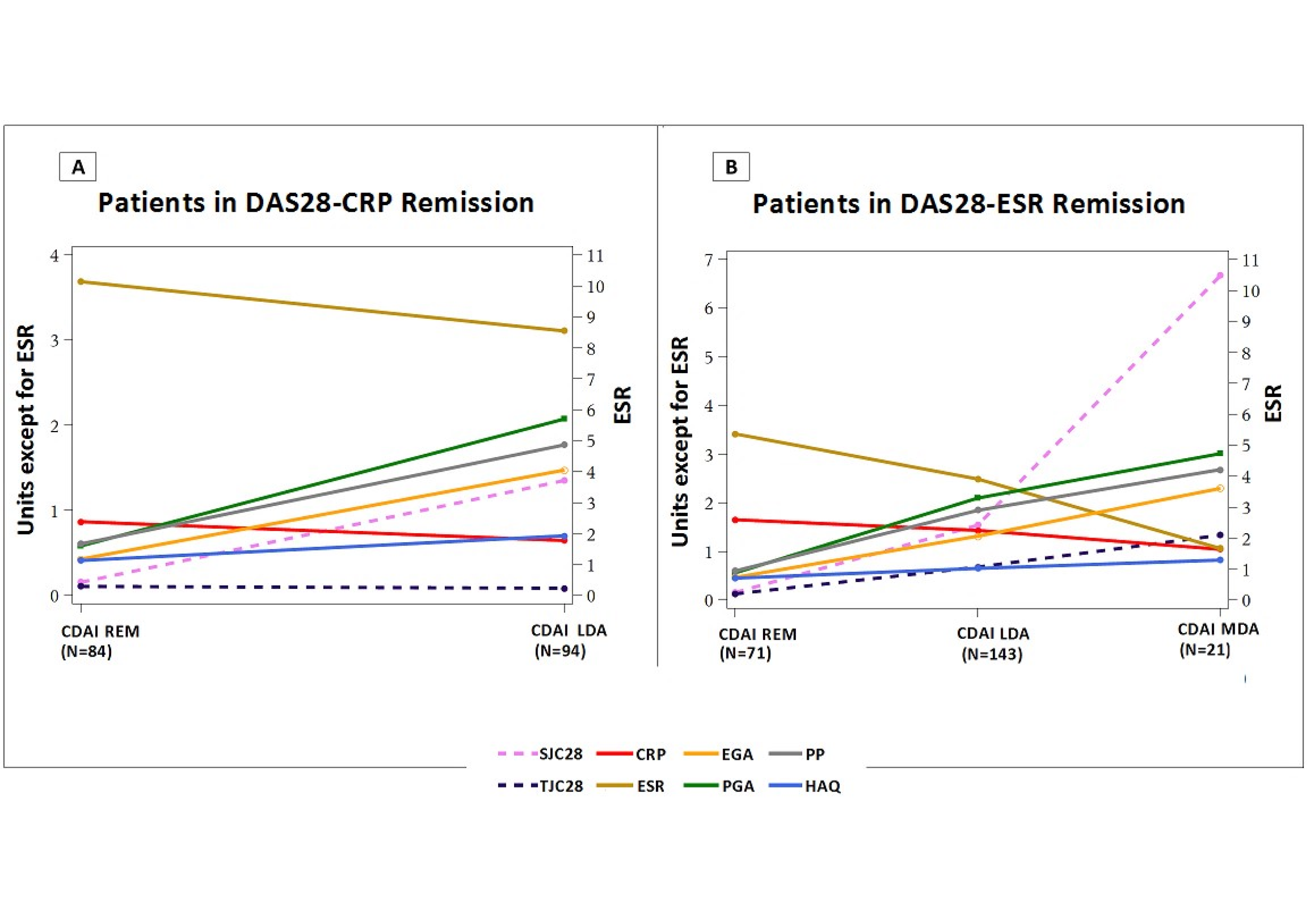

Supplement: Supplementary file 2 — Figure S1. Display of individual disease activity parameters among patients with DAS28-CRP<1.9 who attain different states by CDAI after 24 weeks of tocilizumab therapy. Panel a: patients in remission according to DAS28-CRP classification (n=178). Panel b: patients in remission according to DAS28-ESR classification (n=235). X-axis: CDAI disease activity state. Y-axis: Mean values of respective disease activity parameters. (TIF 2475 kb) [file 13075_2017_1346_MOESM2_ESM.tif]
